# Active cloaking and illusion of electric potentials in electrostatics

**DOI:** 10.1038/s41598-021-89062-1

**Published:** 2021-05-20

**Authors:** Andreas Helfrich-Schkabarenko, Alik Ismail-Zadeh, Aron Sommer

**Affiliations:** 1 University of Applied Sciences, Esslingen, Germany; 2 Institute of Applied Geosciences, Karlsruhe Institute of Technology, Karlsruhe, Germany; 3 Institute of Earthquake Prediction Theory and Mathematical Geophysics, Russian Academy of Sciences, Moscow, Russia; 4 Institute of Information Processing, Leibniz University Hannover, Hannover, Germany

**Keywords:** Solid Earth sciences, Applied mathematics

## Abstract

Cloaking and illusion has been demonstrated theoretically and experimentally in several research fields. Here we present for the first time an active exterior cloaking device in electrostatics operating in a two-horizontally-layered electroconductive domain, and use the superposition principle to cloak electric potentials. The device uses an additional current source pattern introduced on the interface between two layers to cancel the total electric potential to be measured. Also, we present an active exterior illusion device allowing for detection of a signal pattern corresponding to any arbitrarily chosen current source instead of the existing current source. The performance of the cloaking/illusion devices is demonstrated by three-dimensional models and numerical experiments using synthetic measurements of the electric potential. Sensitivities of numerical results to a noise in measured data and to a size of cloaking devices are analysed. The numerical results show quite reasonable cloaking/illusion performance, which means that a current source can be hidden electrostatically. The developed active cloaking/illusion methodology can be used in subsurface geo-exploration studies, electrical engineering, live sciences, and elsewhere.

## Introduction

Invisibility has been a subject of human fascination for millennia. The basic idea of invisibility is to generate a cloaking device and use it to hide an object. Cloaking devices employ specially designed structures that would make objects ‘invisible’ by detecting devices (e.g. eyes, antennas, airborne or satellite detectors/sensors). Over the last two decades, theoretical and experimental studies on cloaking have been conducted in several research fields such as electromagnetism^1,2^, thermal and electrical studies^3^, thermodynamics^4–7^, solid mechanics^8^, acoustics^9–12^, elastic^13,14^, and seismic wave propagation^16–18^.

Cloaking devices can differ by its construction (*interior* and *exterior cloaking*) and by transforming physical properties of the material surrounding an object (*passive cloaking*) or adding an active source (*active cloaking*). An *interior cloaking device* surrounds an object to be cloaked, so that, the object is located in the interior of the cloaking device^10^. An *exterior cloaking device* hides objects from potential detections without encompassing them^19^. A *passive* cloaking device induces invisibility by a special choice of physical parameters of a designed artificial material (so-called *metamaterial*) surrounding or partly surrounding an object, so that, an incident wave on the object bypasses it without distortions. A mathematical technique used to develop metamaterials is transformation optics^20–22^. In the case of electrostatics, such metamaterial would be a material with an anisotropic electrical conductivity^23^. An *active cloaking* masks (emitting) objects using active sources^2,7,24–28^.

**Figure 1:**
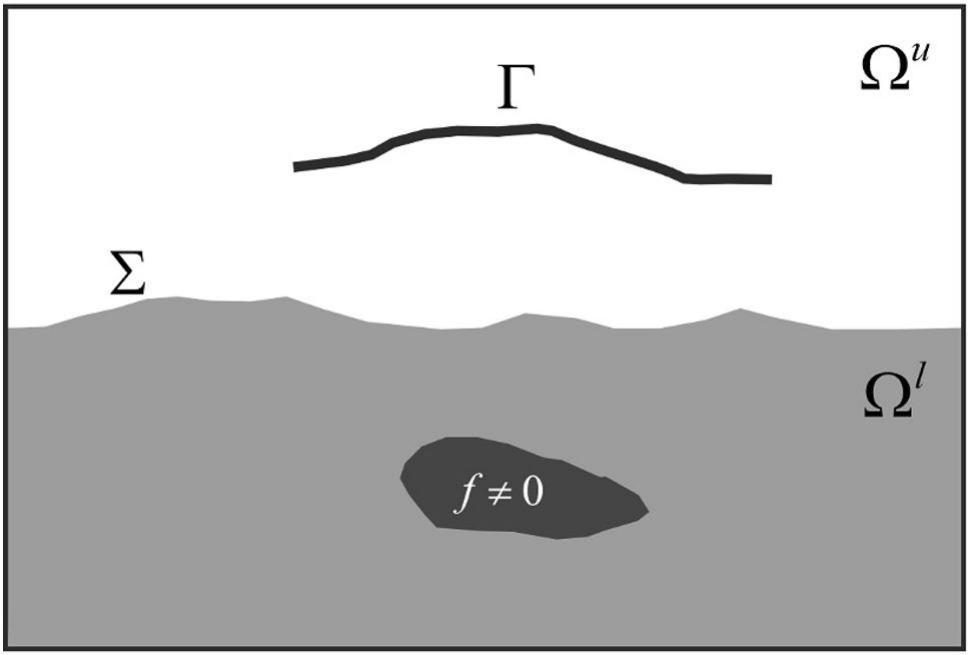
Two-dimensional cartoon of the model domain $\Omega$. Dark gray: the area of non-zero current source density; light gray: the SSD $\Omega^{l}$; $\Sigma$ is the ISD; $\Omega^{u}$ is the OSD; and $\Gamma$ is a curve (or a set) of measurement points.

In this paper, in horizontally-layered electroconductive domain we use active exterior cloaking devices in the case of electrostatics to mask current source located in the source sub-domain (SSD), e.g. Earth’s ground, so making the source nearly undetectable by measurements in the observational sub-domain (OSD), e.g., seawater (Fig. 1). An “invisibility” in this case is achieved by using the current source networks suitably constructed on the interface between the two sub-domains (hereinafter referred to as ISD), which cancel (cloak) or generate imaginary (illusion) electric potential in the OSD. A mathematical background for developing the active cloaking devices lies in the theory of inverse problems^29^ with the use of the superposition principle in terms of active noise control or noise cancellation^30,31^. In a three-dimensional model domain comprised of two overlain electroconductive layers, the following direct and inverse problems form essential components of our numerical experiments based on an electrostatic model.

- *Direct Problem*: To find the generated electrical potential in the entire model domain for a given non-zero current source density located in SSD.
- *Source Identification Problem*: To determine this current source density from its electric potential, which can be measured or inferred from measured electromagnetic data in the OSD. As the source identification problem was analysed by Sommer et al. (ref.^32^), here we describe briefly the results of this study. Applications of the source identification problem are numerous; for example, it is the subject of research in volcanology^33,34^ and in geo-explorations^35^.
- *Active Cloaking Problem*: To cloak the current source density so that it gets ‘invisible’ for measurements in the OSD. To achieve it, we introduce an additional current source density (thereafter referred to as *active cloaking device*) on the ISD in order to minimize the total electric potential field in the OSD.
- *Active Illusion Problem:* To generate an illusion in the data measured in the OSD by manipulating the total electric potential field. The manipulation is set up via an additional current source density on the ISD. A similar approach was used in acoustics and electromagnetics^36,37^. Essentially, an active illusion problem is based on an active cloaking problem.

In what comes next, we present results of the four interconnected problems mentioned above. Synthetic data (that is, an electric potential) are generated by solving the direct problem (hereinafter we refer to the synthetic data as “measured” data). These data are employed as the input data in the source identification problem to determine the current source density. The active cloaking and illusion devices are then introduced to mask the current source, and the effectiveness of the devices is demonstrated.

## Results

### Electric potential determination

The electric potential *u* (measured in V) is determined from the volumetric current source density $f \ne 0$ (measured in A m^−3^; also known as the self-potential source^38,39^) by solving the boundary value problem for the conductivity equation

1 $$- \nabla \cdot (\sigma ({\mathbf{x}})\nabla u({\mathbf{x}})) = f({\mathbf{x}}),\,\,{\mathbf{x}} \in \Omega$$

with the Robin condition at the boundary of the model domain^40^

2 $$\sigma ({\mathbf{x}})\frac{{\partial u({\mathbf{x}})}}{{\partial {\mathbf{n}}({\mathbf{x}})}} + g({\mathbf{x}})u({\mathbf{x}}) = 0,\;\,\,{\mathbf{x}} \in \partial \Omega .$$

Here $\sigma$ is the electrical conductivity (measured in ${\text{S m}}^{ - 1}$); ${\mathbf{x}} = (x_{1} ,x_{2} ,x_{3} )^{T}$ are the Cartesian coordinates; $\Omega ={\Omega }^{l}\cup\Sigma \cup {\Omega }^{u}\subset {\mathbb{R}}^{3}$ is the three-dimensional model domain (its description can be found in *Method*, and its two-dimensional sketch in Fig. 1); ${\Omega }^{l}$ is the SSD, ${\Omega }^{u}$ is the OSD, $\Sigma$ is the ISD; ${\mathbf{n}}$ is the outward unit normal vector at a point on the boundary $\partial \Omega$, which restricts ${\mathbb{R}}^{3}$ to a bounded domain $\Omega$; $\frac{\partial u}{{\partial {\mathbf{n}}}}$ is the normal derivative of the electric potential *u;* and $g$ is a non-negative function defined at the model boundary as the reciprocal distance from the boundary to the geometrical centre of the model domain $\Omega$.

**Figure 2:**
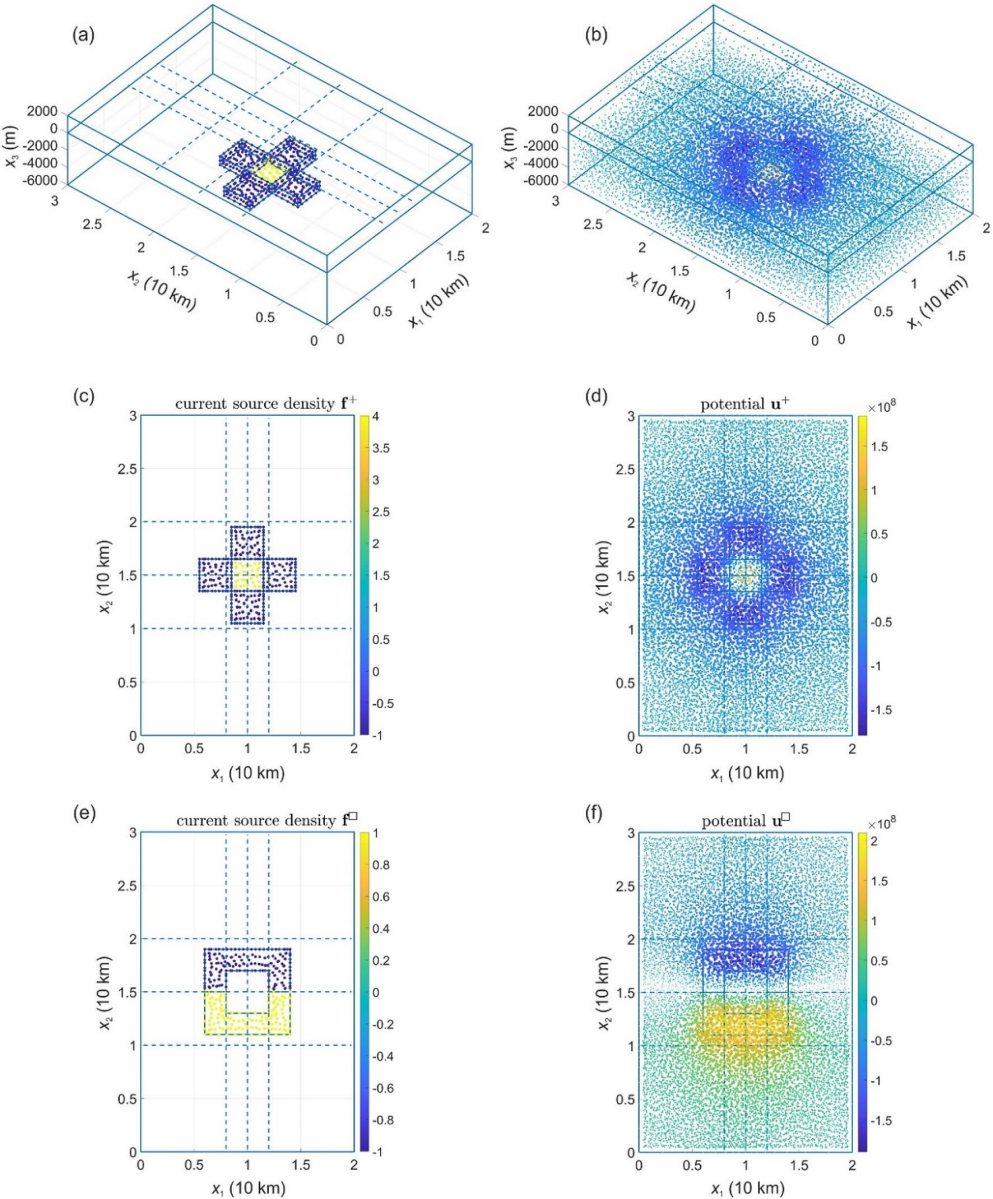
Electric potentials generated by two current source densities. The perspective view (**a**) and top view (**c**) of the current source density ${\mathbf{f}}^{ + }$; the perspective view (**b**) and top view (**d**) of the electric potential ${\mathbf{u}}^{ + }$ generated by the current source density. Top view of the electric potential ${\mathbf{u}}^{ \square }$ (**f**) generated by ${\mathbf{f}}^{ \square }$ (**e**). Here and in Figs. 3, 4, 5, 6, a top view image presents a transparent projection of physical quantities at finite element nodes on the plane. The size of the nodes in the images is proportional to the absolute value of the physical quantities it represents, i.e. the nodes with zero-values are not displayed. Dashed lines show the position of the paths, along which synthetic measurements of the electric potential have been made.

To solve the problem (1)–(2) numerically, the finite-element method is used^41,42^. The solution to a discrete problem corresponding to the weak formulation of the problem (1)–(2) can be presented as:

3 $${\mathbf{u}} = {\mathbf{Af}},$$

where **u** and **f** are the discrete representations of the electric potential and the current source density, respectively, and **A** is the solver operator (see *Method*). The solutions ${\mathbf{u}}^{ + }$ and ${\mathbf{u}}^{ \square }$ for two different current source densities ${\mathbf{f}}^{ + }$ and ${\mathbf{f}}^{ \square }$, respectively (see *Method* for description of the current source densities), are illustrated in Fig. 2. As measurements of the electric potential are restricted to a part of $\Omega^{u}$ (OSD), we introduce the restriction operator ${\mathbf{M}}$, which restricts ${\mathbf{u}}$ to the measured data ${\mathbf{u}}_{d} : = \left. {{\mathbf{Mu}}: = {\mathbf{u}}} \right|_{\Gamma }$, where ${{\varvec{\Gamma}}} \subset {{\varvec{\Omega}}}^{u}$ is a set of measurement points; ${\mathbf{M}}$ is the squared matrix consisting of 0 (no measurement) or 1 (a measured value of **u** exists); and the notation $A: = B$ means that *A* equals *B* by a definition. Using Eq. (3), we obtain:

4 $${\mathbf{u}}_{d} = {\mathbf{MA f}} = :{\mathbf{A}}_{d} {\mathbf{f}}.$$

### Current source identification

The current source density ${\mathbf{f}}$ can be formally determined from the measurements of the electric potential ${\mathbf{u}}_{d}$ by solving Eq. (4), namely, ${\mathbf{f}} = {\mathbf{A}}_{d}^{ - 1} {\mathbf{u}}_{d}$. Meanwhile, it is shown that a solution of this inverse problem exists, but it is not unique^32^. A Tikhonov regularization can enforce the uniqueness of a solution via a spectral shift by using the operator ${\mathbf{A}}_{d}^{{\text{T}}} {\mathbf{A}}_{d}$, where ${\mathbf{A}}_{d}^{\rm T}$ is the transpose of operator ${\mathbf{A}}_{d}^{{}}$^29,43^. Doing so, the following solution to the regularized inverse problem for given measurements ${\mathbf{u}}_{d}$ can be obtained:

5 $${\mathbf{f}}_{\alpha } : = {{\varvec{\Lambda}}}_{\alpha } {\mathbf{u}}_{d} ,\;{{\varvec{\Lambda}}}_{\alpha } = ({\mathbf{A}}_{d}^{\rm T} {\mathbf{A}}_{d} + \alpha {\mathbf{D}}^{{\text{T}}} {\mathbf{D}})^{ - 1} {\mathbf{A}}_{d}^{\rm T} ,$$

where $\alpha > 0$ is the regularization parameter, ${\mathbf{D}}^{{\text{T}}} {\mathbf{D}}$ is the penalty term, and **D** is the discrete Nabla ($\nabla$) operator. As the choice of $\alpha$ is critical in the Tikhonov regularization method, we apply the *L*-curve criterion to find the optimal value of the regularization parameter^44^.

**Figure 3:**
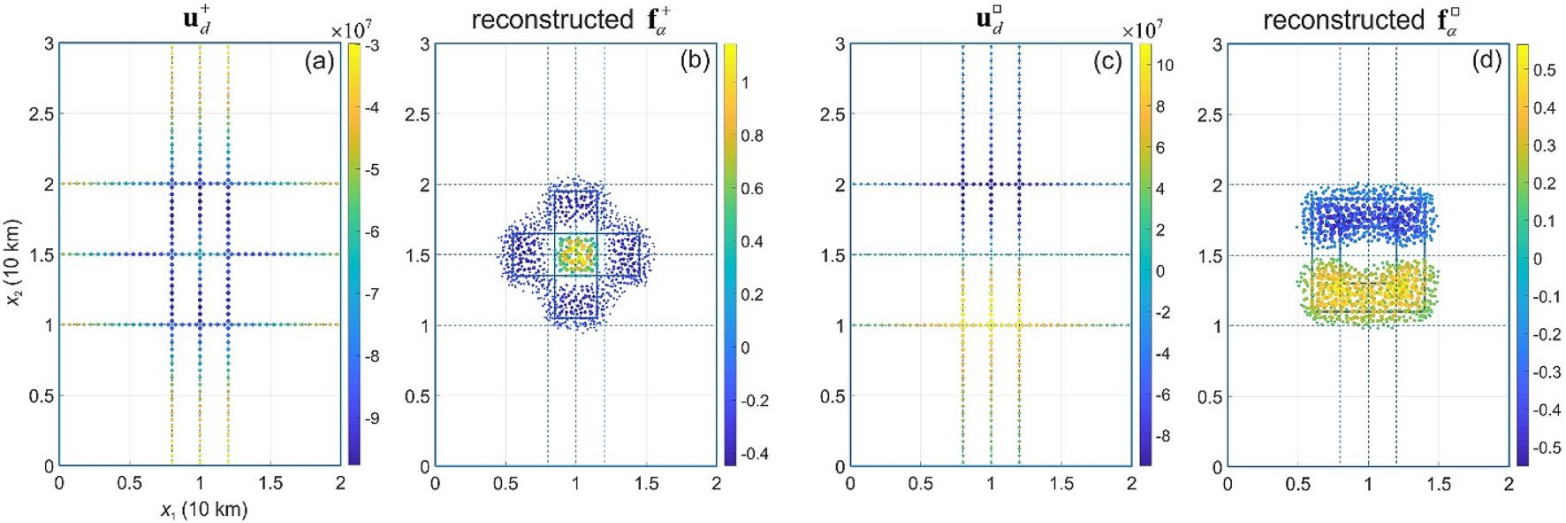
Electric potential $\, {\mathbf{u}}_{d}^{ + }$ (**a**) and ${\mathbf{u}}_{d}^{ \square }$ (**c**) along the paths of synthetic measurements, and the current source density $\, {\mathbf{f}}_{\alpha }^{ + }$ (**b**) and ${\mathbf{f}}_{\alpha }^{ \square }$ (**d**) reconstructed from the synthetic measured data.

The inverse problem (Eq. 5) is solved numerically using the same current source densities ${\mathbf{f}}^{ + }$ and ${\mathbf{f}}^{ \square }$. In our numerical experiments, the set ${{\varvec{\Gamma}}}$ consists of 300 synthetic measurement points located in the OSD along three lines at the height of 500 m (parallel to *x*_1_-axis) and three lines at the height of 1000 m (parallel to *x*_2_-axis) above the plane *x*_3_ = 0 (Fig. 3 a,c). When choosing the points one should ensure that they are distributed rather uniformly in the sub-domain OSD (both in horizontal and vertical dimensions) to blanket the electric current source. This allows for better reconstructing the source density from measurement data, as the source detection power decreases with increasing distance. The data determined on ${{\varvec{\Gamma}}}$ are used to reconstruct $\, {\mathbf{f}}_{\alpha }^{ + }$ and ${\mathbf{f}}_{\alpha }^{ \square }$ as shown in Eq. (5), and the inversion’s results are shown in Fig. 3b,d. The performance of regularization and the sensitivity of numerical results have been tested by introducing a random noise on measurements ${\mathbf{u}}_{d}$. It is shown that the quality of the reconstructions of the current source density decreases with the noise (see *Supplementary Material;* Fig. S1).

### Active cloaking

Here we present an active cloaking device allowing the signals emanating from the electric current source to be cancelled to a considerable extent in the OSD. The active cloaking device means physically a network of electrodes installed on the ISD (although the electrode’s installation can be done everywhere), which produces a complementary electric current source density patters **f**_*c*_ (${\mathbf{f}} + {\mathbf{f}}_{c} \ne 0$), so that, the superimposed signals from the source **f** and from those from the electrodes **f**_*c*_ cancel each other. The electrodes should be distributed on the ISD such a way to blanket the current source to be hidden (the influence of the position of the cloaking device and its size on cloaking is discussed later and in *Supplementary Materials*).

To determine the effective current source density pattern **f**_*c*_, we employ the superposition principle. As the inverse problem associated with the active cloaking device is linear, the superposition principle can be applied. In doing so, the total electrical potential field vanishes on ${{\varvec{\Gamma}}}$ (measurement paths), reduces in the OSD significantly, and hence becomes almost undetectable by measurements. Applying the operator **A**_*d*_ to the combined electric current source density, we obtain

6 $${\mathbf{A}}_{d} \left( {{\mathbf{f}} + {\mathbf{f}}_{c} } \right) = {\mathbf{A}}_{d} {\mathbf{f}} + {\mathbf{A}}_{d} {\mathbf{f}}_{c} = {\mathbf{u}}_{d} + {\mathbf{A}}_{d} {\mathbf{f}}_{c} = 0,$$

i.e. ${\mathbf{A}}_{d} {\mathbf{f}}_{c} = - {\mathbf{u}}_{d}$. The cloaking procedure is described as7
and hence  on ${{\varvec{\Gamma}}}$. Here ${{\varvec{\Lambda}}}_{c,\alpha }$ is the cloaking operator; ${\mathbf{f}}_{c,\alpha }$ is the current source density of the cloaking device; ${\mathbf{A}}_{d,c}$ is the adapted operator, which maps the cloaking current source density ${\mathbf{f}}_{c,\alpha }$ to electrical potential ${\mathbf{u}}_{d,c}^{{}}$ on ${{\varvec{\Gamma}}}$; and the notation  means ${\mathbf{\Phi w}} = {\mathbf{h}}$ (see *Method* for detail, where the cloaking and adapted operators are presented).

**Figure 4:**
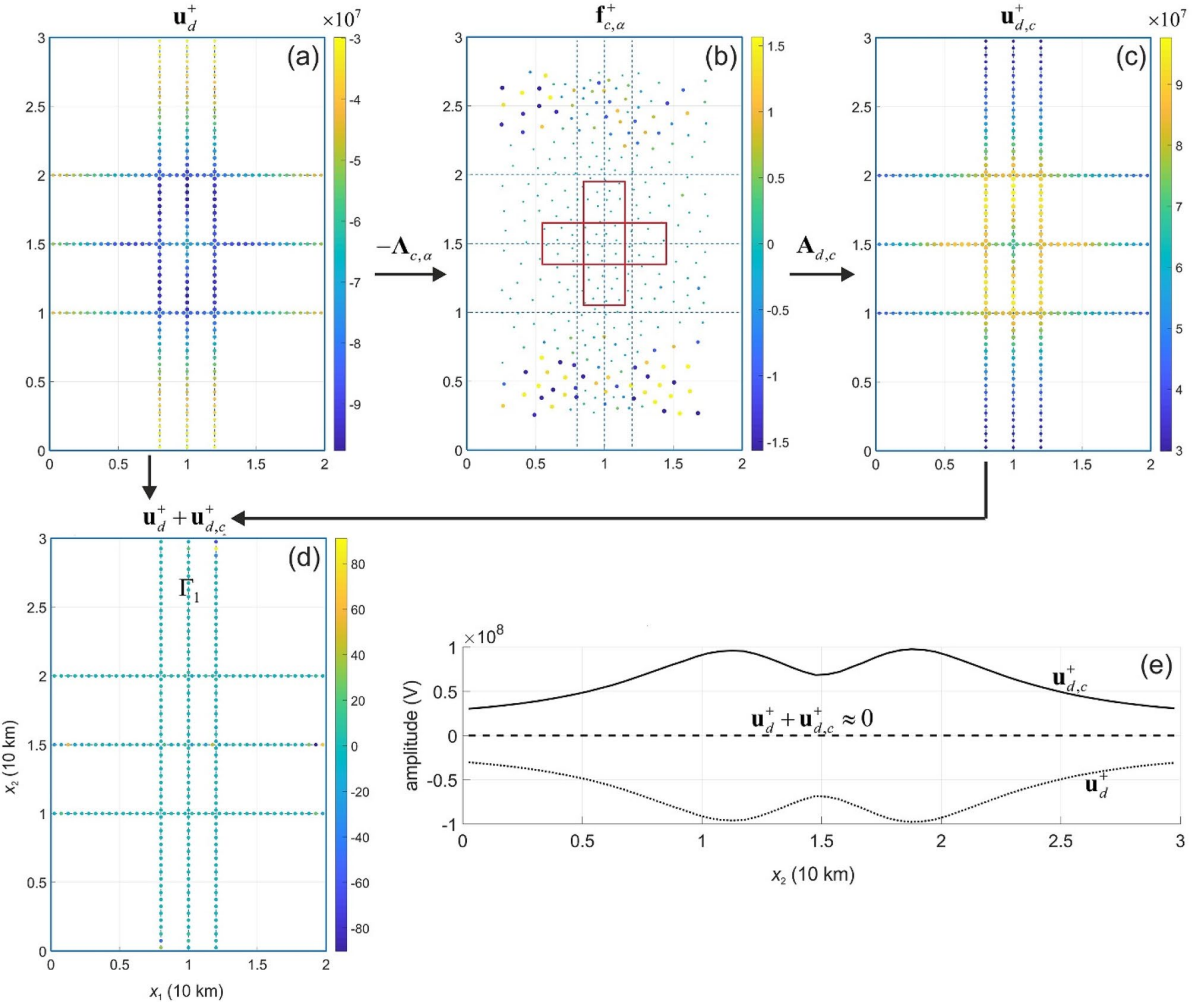
Active cloaking of the electric potential ${\mathbf{u}}_{d}^{ + }$ on ${{\varvec{\Gamma}}}$ (**a**) generated by the current source density ${\mathbf{f}}^{ + }$. Using Eq. (13) the cloaking devise is modelled by the current source density ${\mathbf{f}}_{c,\alpha }^{ + }$ (**b**) that generates ${\mathbf{u}}_{d,c}^{ + }$ (**c**) leading to a significant reduction (almost cancellation) of the electric potential signal on ${{\varvec{\Gamma}}}$ (**d**). Panel (**e**) demonstrates the cancellation of the signal ${\mathbf{u}}_{d}^{ + } + {\mathbf{u}}_{d,c}^{ + }$ (see dashed line) on the middle path (line ${{\varvec{\Gamma}}}_{1}$: $\{ {\mathbf{x}} \in \Omega^{u} :x_{1} = 0{\text{ km}}; \, x_{3} = 0.5{\text{ km}}\} \subset {{\varvec{\Gamma}}}$) of the synthetic measurement data.

**Figure 5:**
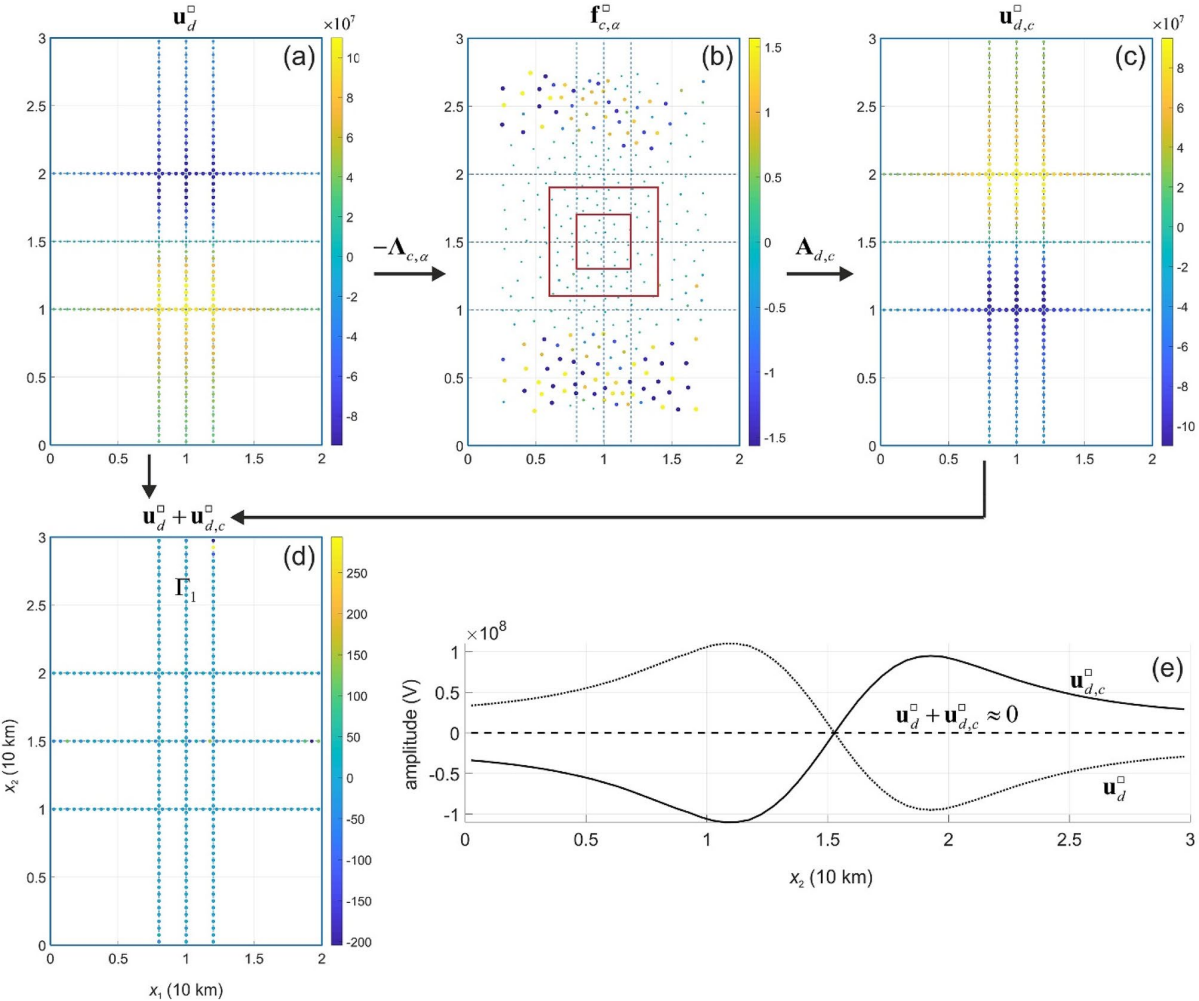
Active cloaking of the electric self-potential ${\mathbf{u}}_{d}^{ \square }$ on ${{\varvec{\Gamma}}}$ (**a**) generated by the current source density ${\mathbf{f}}^{ \square }$. Using Eq. (13) the cloaking devise is modelled by the current source density ${\mathbf{f}}_{c,\alpha }^{ \square }$ (**b**) that generates ${\mathbf{u}}_{d,c}^{ \square }$ (**c**) leading to a significant reduction (almost cancellation) of the electric potential signal on ${{\varvec{\Gamma}}}$ (**d**). Panel (**e**) demonstrates the cancellation of the signal ${\mathbf{u}}_{d}^{ \square } + {\mathbf{u}}_{d,c}^{ \square }$ (see dashed line) on line ${{\varvec{\Gamma}}}_{1}$ $\{ {\mathbf{x}} \in \Omega^{a} :x_{1} = 0{\text{ km}}; \, x_{3} = 0.5{\text{ km}}\} \subset {{\varvec{\Gamma}}}$.

In numerical experiments, we consider the current source densities ${\mathbf{f}}^{ + }$ and ${\mathbf{f}}^{ \square }$ and apply the cloaking operator ${{\varvec{\Lambda}}}_{c,\alpha }$ to synthetic data ${\mathbf{u}}_{d}^{ + }$ and ${\mathbf{u}}_{d}^{ \square }$. The cloaking current pattern ${\mathbf{f}}_{c,\alpha }^{ + }$ and ${\mathbf{f}}_{c,\alpha }^{ \square }$ are presented in Figs. 4 and 5, respectively. Comparing the images of ${\mathbf{A}}_{d} {\mathbf{f}}^{ + }$(Fig. 4a) and ${\mathbf{A}}_{d,c} {\mathbf{f}}_{c,\alpha }^{ + }$ (Fig. 4c), we see that the images are almost identical up to their sign, and their sum is almost vanishing (Fig. 4d). The cloaking operator ${{\varvec{\Lambda}}}_{c,\alpha }$ significantly reduces the amplitude of the total electric potential from about 10^8^ to 10^2^ V (Fig. 4b,e). Similarly, the operator ${{\varvec{\Lambda}}}_{c,\alpha }$ reduces the amplitude of the total electric potential in the case of the current source $\, {\mathbf{f}}^{ \square }$ (Fig. 5). Figures 4e and 5e illustrate the cancellation of the signals ${\mathbf{u}}_{d}^{ + } + {\mathbf{u}}_{d,c}^{ + }$ and ${\mathbf{u}}_{d}^{ \square } + {\mathbf{u}}_{d,c}^{ \square }$, respectively, where the dashed line represents the total electric potential field. The cloak regime masks the source for measurements, and, therefore, the current source becomes invisible electrostatically, i.e. cloaked. Note that the cloaking device (i.e. electric current source density ${\mathbf{f}}_{c,\alpha }$; Figs. 4b and 5b) was designed based on data ${\mathbf{u}}_{d}$ and not on ${\mathbf{f}}$.

**Figure 6:**
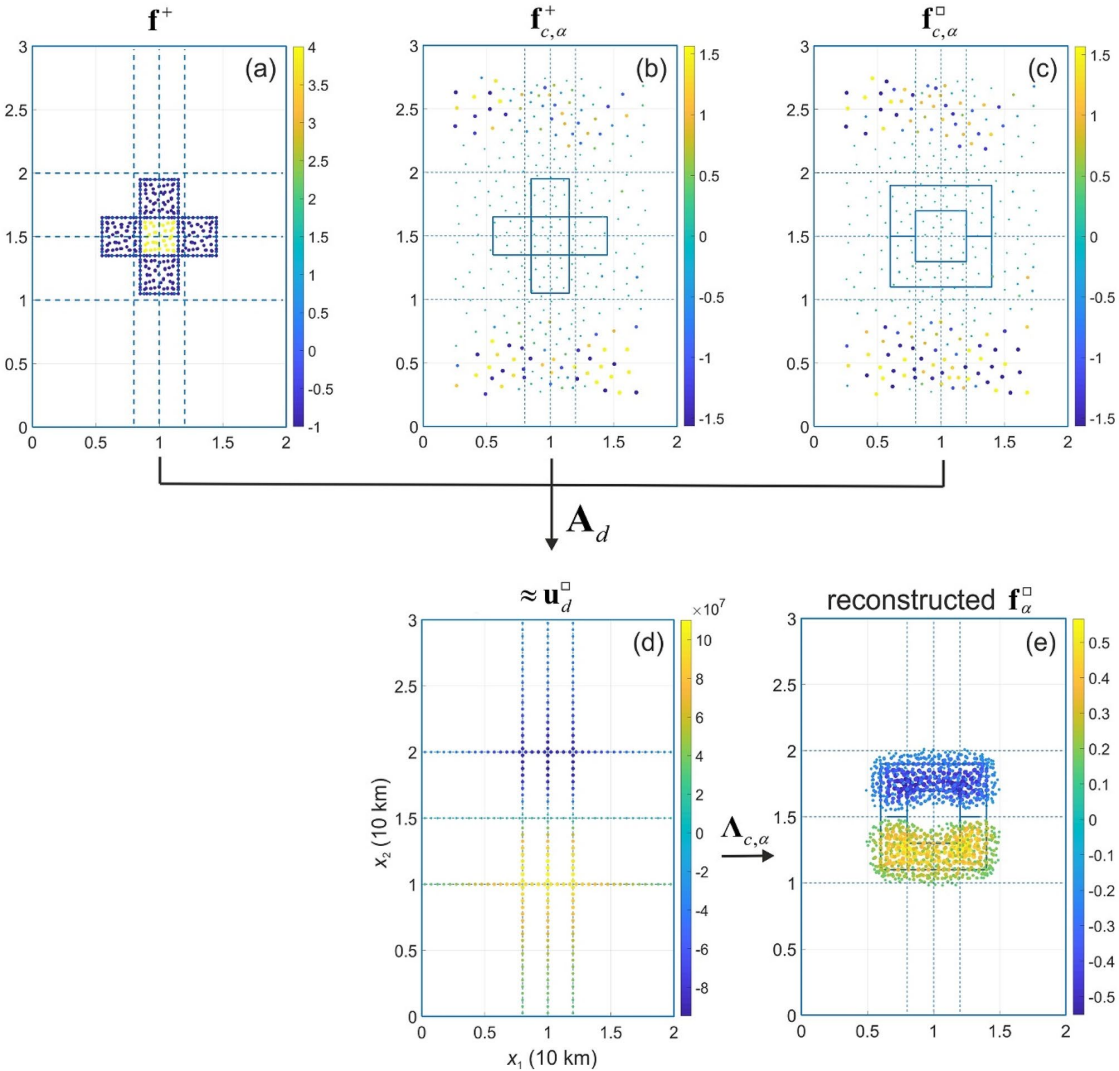
Illusion of the current source density ${\mathbf{f}}_{{}}^{ \square }$ by cloaking of the current source density ${\mathbf{f}}_{{}}^{+ }$ (**a**; see also Fig. 2) using cloaking devices ${\mathbf{f}}_{c,\alpha }^{+ }$ (**b**) and ${\mathbf{f}}_{c,\alpha }^{ \square }$ (**c**). Panel (**d**) represents a composition data on electric potential, which will be measured in the case of illusion (Eq. 16), and panel (**e**) illustrates the reconstruction of current source density from these measured data.

In the numerical experiments presented here, the position and size of the cloaking device on the ISD have been fixed. To what extent do its position and size affect the cloaking? To answer the questions, we have performed several numerical experiments (see *Supplementary Materials)*. It is shown that the accuracy of the devices enhances with the increasing size of the devices (Fig. S2). A shift of the cloaking devices may improve the quality of invisibility (Figs. S3 and S4). Hence, a search for the optimal size and the position of a cloaking device will assist in enhancing invisibility.

When developing the cloaking device, we have considered synthetic data of the electric potential along several paths in the OSD, i.e. the cloaking device ensures that the electric potential becomes insignificant (invisible) on the paths. Meanwhile, how would the cloaking device look like and how effective would it be, if we use not only these paths to develop the cloaking device, but the entire OSD? To ensure the invisibility of the current source everywhere in the OSD (not only along the paths of measurements), a cloaking device has been developed based on the measurements in the entire OSD. It is shown that although the quality of the cloaking lowers in this case, but still reducing the signal of electric potential by an order of magnitude (Fig. S5).

### Active illusion

An illusion is generated in numerical experiments such a way that measurements in the OSD “detect” a current source artificially constructed instead of the existing current source located in the SSD. We achieve this by introducing a specially-designed illusion device, which, according to the principle of superposition, changes the total electric potential field in $\Omega^{u}$ into that generated by the current source density chosen for the illusion.

For given ${\mathbf{f}}^{ + }$ in the SSD, we determine an additional current source density ${\mathbf{f}}_{i} = {\mathbf{f}}_{c}^{ + } - {\mathbf{f}}_{c}^{ \square }$ on the ISD so that the inverse problem approach applied to the new data ${\mathbf{u}}_{d}^{ + } + {\mathbf{A}}_{d} {\mathbf{f}}_{i}$ delivers a solution corresponding to ${\mathbf{f}}_{\alpha }^{ \square }$. Namely,

8 $$\begin{aligned} {\mathbf{A}}_{d} ({\mathbf{f}}^{ + } + {\mathbf{f}}_{i} ) = & {\mathbf{A}}_{d} ({\mathbf{f}}^{ + } + {\mathbf{f}}_{c}^{ + } - {\mathbf{f}}_{c}^{ \square } ) = {\mathbf{A}}_{d} {\mathbf{f}}^{ + } + {\mathbf{A}}_{d} {\mathbf{f}}_{c}^{ + } - {\mathbf{A}}_{d} {\mathbf{f}}_{c}^{ \square } \\ = & \underbrace {{{\mathbf{u}}_{d}^{ + } + {\mathbf{u}}_{d,c}^{ + } }}_{\begin{subarray}{l} \approx 0{\text{ (Eq}}{. 13 } \\ {\text{in }}Method{)} \end{subarray} } - {\mathbf{u}}_{d,c}^{ \square } \approx - {\mathbf{u}}_{d,c}^{ \square } = - {\mathbf{A}}_{d} {\mathbf{f}}_{c}^{ \square } \approx {\mathbf{u}}_{d}^{ \square } . \\ \end{aligned}$$

This means that the illusion pattern ${\mathbf{f}}_{i}$ generates “measured” data ${\mathbf{u}}_{d}^{ \square }$ corresponding to ${\mathbf{f}}_{{}}^{ \square }$. The current source density ${\mathbf{f}}_{{}}^{ \square }$ has been chosen just for simplicity of the illustration of the illusion’s results; any admissible current source density can be considered as an additional source.

The illusion procedure can be briefly described as  , where the cloaking patterns ${\mathbf{f}}_{c,\alpha }^{ + }$ and ${\mathbf{f}}_{c,\alpha }^{ \square }$ are determined from data ${\mathbf{u}}_{d}^{ + }$ and ${\mathbf{u}}_{d}^{ \square }$. Assuming that the given current source density is ${\mathbf{f}}^{ + }$ (Fig. 6a), the cloaking pattern ${\mathbf{f}}_{c,\alpha }^{ + }$ (Fig. 6b) and ${\mathbf{f}}_{c,\alpha }^{ \square }$ (Fig. 6c) are computed. Then the operator ${\mathbf{A}}_{d}^{{}}$ (Eq. 8) is applied to the current source density ${\mathbf{f}}^{ + } + {\mathbf{f}}_{c,\alpha }^{ + } - {\mathbf{f}}_{c,\alpha }^{ \square }$ to get the resulting “measured” data ${\mathbf{u}}_{d}^{ \square }$ (Fig. 6d). Finally, applying the cloaking operator ${{\varvec{\Lambda}}}_{c,\alpha }$ the illusive current source density ${\mathbf{f}}_{\alpha }^{ \square }$ is obtained (Fig. 6e).

## Discussion

In this work, an approach to design exterior active cloaking devices for self-potentials is presented, and it has been applied to an electrostatic problem so that an electric current source located in the SSD becomes “undetectable” by measurements in the OSD. Compared to the passive cloaking devices, active devices are more simpler as they do not need metamaterials to be constructed and employed. Using synthetic examples of electric current sources, we have obtained that a constructed camouflage on the ISD allows to reduce significantly (at least by six orders of magnitude) the signal of the electrical self-potential on given measurement paths in the OSD, which is emanated from the electric current source located in the SSD. We note that the same approach can be applied to develop interior active cloaking devices by specifying the support of ${\mathbf{f}}_{c}$ around the source to be hidden, i.e., the cloaking device envelops the source completely.

Although the results of the study are promising and show that the amplitude of the total electric potential is reduced by several orders of magnitude compared to the measured data, a full cloaking cannot be reached due to several reasons. An exterior cloaking device considered here does not envelop completely the source to be hidden. The smaller is the size of the cloaking device, the less effective it is (see Fig. S2 in Supplementary Material). Similarly, the effectiveness of the cloaking device will depend on the network of electrodes installed on the ISD: the denser network, the better results. However, a computational cost will increase with the denser network of electrodes associated with computational nodes. Moreover, the regularization of inverse problems as well as numerical errors degrade the quality of cloaking.

In addition, we have extended the idea of cloaking in electrostatic problems to illusion by manipulating the cloaking device so that the observed field of electric self-potential contains a superposition of hidden field created by the electric source in the SSD and a completely new field, which can be generated arbitrarily. Using synthetic examples, we have demonstrated the applicability of the illusion approach to the same electrostatic problem and shown that a “cross”-type source in the SSD becomes invisible, but instead a “ring”-type source can be reconstructed from measurements in the OSD. Since it is more difficult to make an object completely invisible/undetectable due to the measurement inaccuracy and noise, an illusion device can help to hide a real shape of the source or object by mimicking another modelled shape. For example, a source or object could become smaller or bigger for an observer, like a transformation of the ogre into a lion and a mouse in the fairy tale *Puss in Boots* by Charles Perrault.

Electric self-potentials are usually generated by a number of natural sources, such as electrochemical, electrokinetic, thermoelectric, and mineral sources, as well as by a conducting fluid flow through the rocks. Self-potentials can fluctuate in the Earth with time due to different processes, e.g., alternating currents induced by effects of thunderstorms or heavy rainfalls; variations in Earth’s magnetic fields^45^. As hydrocarbons in a reservoir are moving continuously because of stress and pressure differences, seismic or other vibrations, they create alterations in the electric potentials acting as an electric dipole in the geo-electromagnetic field^46,47^.

Non-invasive measurements of self-potential in the subsurface does not require electric currents to be injected into the ground as in the cases of resistivity or induced polarisation tomography. The method has been used in geological explorations^48^ to detect massive ore bodies, in groundwater and geothermal investigations, environmental and engineering applications, to monitor a salt plume, volcano and lava dome activities, and to reveal a borehole leak during hydraulic fracturing^49–51^. Airborne or seaborne geophysical surveying allows for detecting changes in physical variables of sub-surface processes in the Earth, e.g., in the electromagnetic potential and electric conductivity^52^. The surveying has been used for subsurface exploration, such as hydrocarbon exploration, groundwater management, and shallow drilling hazards.

The presented methods of cloaking and illusion can be used in geo-exploration. For example, depending on a commercial confidentiality, operators may wish to cloak the subsurface objects in electrostatic sense from airborne/seaborne measurements by other operators. We note that when the OSD is filled by seawater, an electric potential can be measured by seaborne surveys. Meanwhile, during airborne prospecting, a measured value is the amplitude of the magnetic field. This amplitude can be then converted into an electric potential using an appropriate operator, such that the presented approach based on the Tikhonov regularization can be applied^32^. The airborne/seaborne surveying provides the information on aquifers for groundwater investigations, paleochannels for shallow gas investigation and drilling hazards, on soils and overburden for engineering purposes^53,54^. Cloaking and illusion can be used in these studies as well, depending on purposes and needs of subsurface explorations.

The superposition principle in terms of active noise cancellation presented here can be used in other areas, e.g. in submarine engineering and marine research. The corrosion of a submarine may create an underwater electric potential that can be detected by available seabed mines with appropriated sensors^55^. The cancellation of the underwater electric potential could be improved by using the presented approach. Also, there are living creatures perceiving electric or electromagnetic signals, and this behaviour of the creatures is an important component of their survival strategy. For example, the Gnathonemus elephantfish, hammerhead shark and platypus rely on their electric receptors in muddy waters rather than on their optic sensory organs^56–58^. So, to hide objects from hammerhead sharks, a cloaking or deflecting device could be developed. We believe that an active cloaking and illusion in electrostatics will inspire new applications in geosciences, electrical engineering, live sciences, and elsewhere.

## Method

We employ a weak formulation of the boundary value problem (Eqs. 1 and 2) transforming it into an integral equation:

9 $$B(u,v) = L(f,v),$$

where the operators *B* and *L* are defined as $$B(u,v): = \, \int_{\Omega } {\sigma (x)\nabla u(x) \cdot \nabla v(x)} \, dx$$$$+ \int_{\partial \Omega } {g(x)u(x)v(x)} \, dS$$ and $$L(f,v): = - \int_{\Omega } {f(x)v(x)dx}$$. Here *v* is the test function, and *S* is the boundary element. The solution to the problem (9) for given $$\sigma$$ and $$f$$ (the electric potential *u*) is the weak solution to the original problem^41,42^ (Eqs. 1 and 2). This solution is unique in the case of sufficiently smooth function $$\sigma$$^59^.

We specify the model domain as a cuboid $\Omega = [0.0, \, 20.0] \times [0.0, \, 30.0] \times [ - 6.0, \, 2.0]$ with length unit km, where SSD is represented as $\Omega^{l} = [0.0, \, 20.0] \times [0.0, \, 30.0] \times [ - 6.0,{ 0}.0]$, OSD as $\Omega^{u} = [0.0, \, 20.0] \times [0.0, \, 30.0] \times [0.0,{ 2}.0]$, and ISD (or $\Sigma$) as $x_{3} = 0$. The finite-element method (FEM) is employed^42^, and the model domain is discretized by tetrahedral finite elements at $n = 18 \times 10^{3}$ nodes. The electric potential $u$ and the test function $v$ are approximated by a combination of $n$ linear finite elements, that is, piecewise linear polynomials, $\left\{ {v_{i} } \right\}_{i = 1}^{n}$, i.e. $u(x): = \sum\nolimits_{i = 1}^{n} {u_{i} v_{i} (x)}$ and ${\mathbf{u}}\left( {u_{1} ,u_{2} , \ldots ,u_{n} } \right)^{{\text{T}}} \in {\mathbb{R}}^{n}$. Inserting the approximation into Eq. (9), we obtain a discrete problem corresponding to the problem (9)

10 $${\mathbf{Bu}} = {\mathbf{Lf}},$$

valid for all $$v_{i}$$$$(i = 1,2,3, \, ... \, ,n)$$ with matrices.

${\mathbf{B}}: = \left\{ {B_{ij} = \int_{\Omega } {\sigma ({\mathbf{x}})\nabla v_{i} ({\mathbf{x}}) \cdot \nabla v_{j} ({\mathbf{x}})} \, d{\mathbf{x}} + \int_{\partial \Omega } {{\text{g}} ({\mathbf{x}})v_{i} ({\mathbf{x}})v_{j} ({\mathbf{x}})} \, dS} \right\} \, \in \, {\mathbb{R}}^{n \times n}$, ${\mathbf{L}}: = \left\{ {L_{ij} = - \int_{\Omega } {f_{i} ({\mathbf{x}})v_{j} ({\mathbf{x}})} \, d{\mathbf{x}}} \right\} \, \in \, {\mathbb{R}}^{n \times n} , \, (i,j = 1,2,...,n)$. The vectors ${\mathbf{u}}$ and ${\mathbf{f}}$ are discrete representatives of the electric potential and the current source density, respectively. Sommer et al.^32^ showed that the numerical direct problem (10) is well-posed, and the operator ${\mathbf{B}}$ is positive definite and invertible. Hence, the solution to (10) is ${\mathbf{u}} = {\mathbf{B}}^{ - 1} {\mathbf{L}} \, {\mathbf{f}} = :{\mathbf{Af}}$. It is important to note that the existence of the forward problem’s solver operator ${\mathbf{A}} = {\mathbf{B}}^{ - 1} {\mathbf{L}}$ and its positive definition^41^ as well as the symmetry and the positive definition of matrix ${\mathbf{L}}$ yield the operator ${\mathbf{A}}$ to be invertible.

At each node of the discrete model domain ${{\varvec{\Omega}}}$, we assume the specific electrical conductivity to be $\sigma = 10^{ - 1}$ S m^−1^ for $x_{3} \le 0$ (in the SSD and on the ISD), and $\sigma = 10^{ - 6}$ S m^−1^ for $x_{3} > 0$ (in the OSD). We consider two examples of artificial current source densities (in A m^−3^):

$${\mathbf{f}}^{ + } ({\mathbf{x}}): = \left\{ {\begin{array}{*{20}l} {4,} \hfill & {{\mathbf{x}} \in Q,} \hfill \\ { - 1,} \hfill & {{\mathbf{x}} \in K\backslash Q,} \hfill \\ {0,} \hfill & {\text{elsewhere,}} \hfill \\ \end{array} } \right.\;{\mathbf{f}}^{ \square } ({\mathbf{x}}): = \left\{ {\begin{array}{*{20}l} {1,} \hfill & {x_{2} \ge 0\;{\text{and}}\;{\mathbf{x}} \in R,} \hfill \\ { - 1,} \hfill & {x_{2} < 0\;{\text{and}}\;{\mathbf{x}} \in R,} \hfill \\ {0,} \hfill & {\text{elsewhere,}} \hfill \\ \end{array} } \right.$$

where

$$Q = [8.5,11.5]\;{\text{km}} \times [13.5,16.5]\;{\text{km}} \times [ - 3.5, - 2.5]\;{\text{km,}}$$

$$\begin{aligned} K = & (([8.5,11.5]\;{\text{km}} \times [10.5,19.5]\;{\text{km}}) \, \\ & \vee ([5.5,14.5]\;{\text{km}} \times [13.5,16.5]\;{\text{km}})) \times [ - 3.5, - 2.5]\;{\text{km ,}} \\ \end{aligned}$$

$$\begin{aligned} R = & (([6.0,14.0]\;{\text{km}} \times [11.0,19.0]\;{\text{km}}) \\ & \backslash ([8.0,{1}2.0]\;{\text{km}} \times [13.0,{17}.0]\;{\text{km}})) \times [ - 3.5, - 2.5]\;{\text{km}}{.} \\ \end{aligned}$$

Note that the support $K$ of ${\mathbf{f}}^{ + }$ is a simply connected domain (a “cross”) and the support $R$ of ${\mathbf{f}}^{ \square }$ is a double connected domain (a “ring”). Function *g* is defined in the model as $g({\mathbf{x}}) = \left( {\left[ {x_{1} - 10{\text{ (km)}}} \right]^{2} + \left[ {x_{2} - 15{\text{ (km)}}} \right]^{2} + \left[ {x_{3} + 2{\text{ (km)}}} \right]^{2} } \right)^{{{{ - 1} \mathord{\left/ {\vphantom {{ - 1} 2}} \right. \kern-\nulldelimiterspace} 2}}}$.

We employ the COMSOL Multiphysics FEM software (www.comsol.com) to generate the mesh. The direct and the inverse problem solvers are implemented in MATLAB (www.mathworks.com), which is linked to COMSOL Multiphysics.

In constructing the cloaking device, we assume that the complementary current source density ${\mathbf{f}}_{c}$ has a support on the ISD, and introduce a continuation operator ${\mathbf{U}}$ extending the support as ${\mathbf{Uf}}_{c} ({\mathbf{x}}) = {\mathbf{f}}_{c} ({\mathbf{x}})$ for ${\mathbf{x}} \in \Sigma$ and ${\mathbf{Uf}}_{c} ({\mathbf{x}}) = 0$ elsewhere in $\Omega$. Thus, the domain of the cloaking device corresponds to the domain of ${\mathbf{f}}_{c}$ and is shaped by ${\mathbf{U}}$. We introduce the adapted operator, which maps the cloaking current source density to the electrical potential in the entire domain $\Omega$: ${\mathbf{A}}_{d,c} : = {\mathbf{A}}_{d} {\mathbf{U}}$. The active cloaking problem is formulated as a minimization problem with a penalty term:

11 $$\frac{1}{2}\left\| {{\mathbf{A}}_{d,c} {\mathbf{f}}_{c} ({\mathbf{x}}) + {\mathbf{u}}_{d} ({\mathbf{x}})} \right\|_{{L_{2} (\Gamma )}}^{2} + \frac{\alpha }{2}\left\| {{\mathbf{Df}}_{c} ({\mathbf{x}})} \right\|_{{L_{2} (\Sigma )}}^{2} \to \mathop {\min }\limits_{{{\mathbf{f}}_{c} }} ,$$

where $L_{2} (G)$ is the space of functions that are square integrable over domain $G$, equipped with the standard scalar product $({\mathbf{u}},{\mathbf{v}}) = \int_{G} {{\mathbf{u}}({\mathbf{x}}){\mathbf{v}}({\mathbf{x}})dG}$ and the norm $\left\| {\mathbf{v}} \right\| = ({\mathbf{v}},{\mathbf{v}})^{1/2}$. The solution to the minimization problem (11) can be found using the Tikhonov regularization in the following form^60^:

12 $${\mathbf{f}}_{c,\alpha } = - {{\varvec{\Lambda}}}_{c,\alpha } {\mathbf{u}}_{d} ,$$

where ${{\varvec{\Lambda}}}_{c,\alpha } = ({\mathbf{A}}_{d,c}^{{\text{T}}} {\mathbf{A}}_{d,c} + \alpha {\mathbf{D}}^{{\text{T}}} {\mathbf{D}})^{ - 1} {\mathbf{A}}_{d,c}^{{\text{T}}}$ is the cloaking operator, and ${\mathbf{f}}_{c,\alpha }$ is the current source density of the cloaking device. We define here the electric potential data on ${{\varvec{\Gamma}}}$ generated by ${\mathbf{f}}_{c,\alpha }$ as

13 $${\mathbf{u}}_{d,c}^{{}} : = {\mathbf{A}}_{d,c} {\mathbf{f}}_{c,\alpha } \approx - {\mathbf{u}}_{d} .$$

The cloaking procedure (7) can be then obtained using Eqs. (12) and (13). As the computational design of the cloaking device is based on a Tikhonov regularization, the quality of numerical results depends on the choice of the regularization parameter $\alpha$, and a search for the suitable parameter $\alpha$ is computationally extensive. The active cloaking problem has been solved for different values of the regularization parameter, and the value providing an optimal cancellation of the electric potential signal on measurement paths has been then chosen.

## Supplementary Information

Supplementary Information

## Data Availability

The codes and datasets generated during the current study are available from the first author on a request.
