# Supplementary material for: Active cloaking and illusion of electric potentials in electrostatics
Source: Sci Rep. 2021 May 20;11:10651. doi: 10.1038/s41598-021-89062-1 (PMC8137708; doi:10.1038/s41598-021-89062-1)
Supplement: Supplementary file 1 — Supplementary Information [file 41598_2021_89062_MOESM1_ESM.pdf]

Supplementary Material

ACTIVE CLOAKING AND ILLUSION OF ELECTRIC POTENTIALS IN ELECTROSTATICS

by Andreas Helfrich-Schkabarenko, Alik Ismail-Zadeh, Aron Sommer

Sensitivity of numerical results with respect to noisy data

An advantage of the Tikhonov regularization is its ability to handle with noisy data by adjusting regularization parameter. As experimental measurements are usually polluted by a noise, we impose a random noise on measurements of the electric potential $\mathbf{u}_d$ as $\mathbf{u}_d^{\delta} = \mathbf{u}_d + \delta\boldsymbol{\gamma}$, where $\boldsymbol{\gamma}$ is a standard Gaussian random generator, and δ is its magnitude scaling the noise level in sense of the signal-to-noise ratio in decibel V (dBV). Note that the higher is the noise, the larger values of the regularizing parameter α has to be chosen based on L-curve criterion (e.g. Kirsch, A. An Introduction to the Mathematical Theory of Inverse Problems, Springer, Berlin, 2011). The performance of regularization to handle the noise is shown in Fig. S1.

Two original electric current sources ($\mathbf{f}^{+}$ and $\mathbf{f}^{\square}$) are presented in the left panels of Fig. S1 (a: "cross"-type source; and f: "ring"-type source). The sources are constructed so that the sum of the positive and negative current source densities be equal to zero. All other panels in Fig. S1 show reconstructed sources based on the electric potential data along six measurement paths for various levels of noises. The second column (panels b and g) shows the reconstruction results from the electric potential data without a noise (these reconstructions are used in this study). The shape of the current source is smeared slightly, and its magnitude reduced by a factor of two. Smearing occurs due to regularization of the ill-posed inverse problem. The third column shows that the smearing of the current source becomes larger for noisy data ($\delta = 30$ dBV). With the increasing noise level, the quality of the reconstruction degrades until the sources become barely recognizable in the results (e.g. for the noise level $\delta = 10$ dBV). Hence we assume that a cloaking device could be designed even at slightly noisy measurement data, and the noise level is to be determined for each individual case.

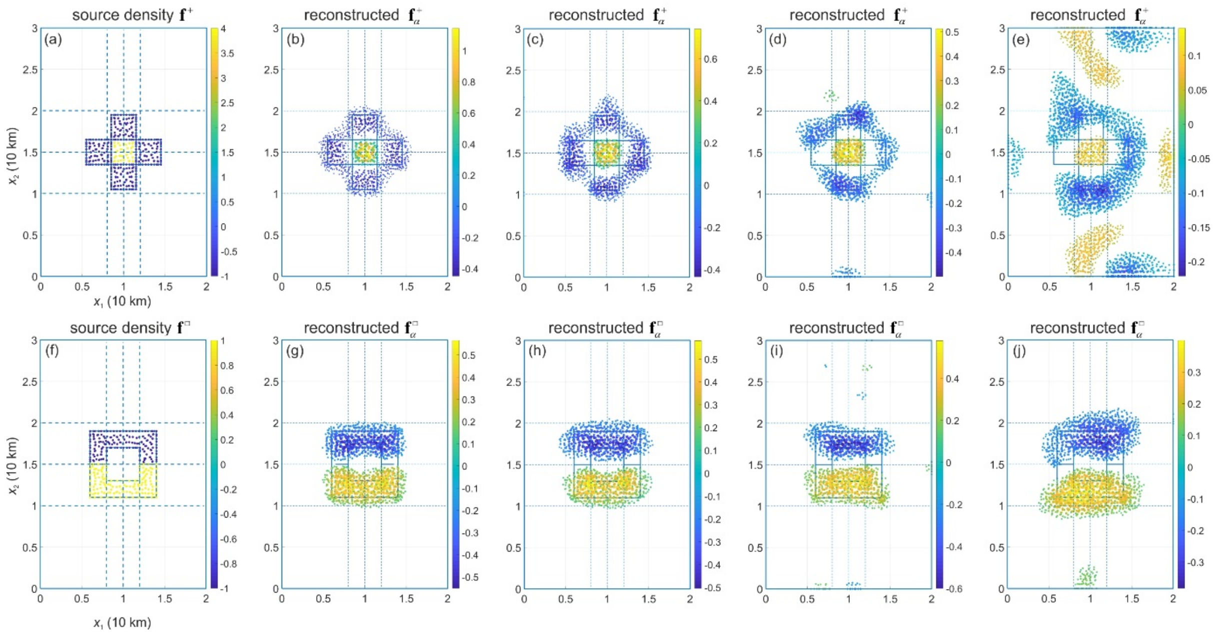

Fig. S1. Reconstruction of the electric source density $\mathbf{f}^{+}$ (a; upper panels) and $\mathbf{f}^{\square}$ (f; lower panels) from the synthetic measurements in case of no noise, $\delta=0$ (b, g) and the noise of δ=30 dBV (c, h), δ=20 dBV (d, i), and δ=10 dBV (e, j).

Sensitivity of numerical results with respect to cloaking device's size and its location

Customising the size of the cloaking device leads to the change in the number of associated finite element (FE) nodes, as we use a fixed FE grid for all experiments. Particularly, the number of FE nodes of the cloaking devices are 120 for the device's size of 15 km x 10 km (Fig. S2a), 170 for the size of 15 km x 15 km (Fig. S2b), 230 for the size of 15 km x 20 km (Fig. S2c), and 300 for the size of 15 km x 25 km (Fig. S2d; the size of the cloaking devise used in the modelling). To evaluate the efficiency of the cloaking device, we consider the value of the electric potential at the middle path in x_2-direction (Fig. S2, e-h). The efficiency of the device increases with the devise's size (i.e. the increasing number of FE nodes). In particular, for the smaller size of the cloaking device, the cloaking operates reasonably well only above the cloaking device (Fig. S2, f).

Figure S3 illustrates the effect of displacement of the cloaking device with respect to the source to be hidden. We consider a cloaking device of size 15 km x 20 km centrally positioned on the ISD (Fig. S3a) and that displaced by 1.5 km in x_2-direction (Fig. S3b). This shift of the cloaking device improves the invisibility as shown in Fig. S3 (c, d). A shift of a smaller size cloaking device could also improve invisibility. For example, if the cloaking device of the size 15 km x 15 km is shifted by a half of its size, the invisibility is improved (Fig. S4, a-d). Therefore, a search for the optimal size and the position of a cloaking device is important to improve invisibility.

The current source density pattern of the cloaking device was computed employing Eq. (11), where the data on electric potential $\mathbf{u}_d$ were measured along a few paths in the OSD (consisting of about 300 FE nodes; see Fig. S5a). To ensure the invisibility of the current source everywhere in Ω^u, $\mathbf{u}_d$ should contain measurements in the entire Ω^u (consisting of about 1200 FE nodes in our numerical experiments). This results in the cloaking device shown in Fig. S5b, which has more homogeneous current source density pattern than that for the cloaking device developed earlier (Fig. S5a; Fig. 4b). The efficiency of the cloaking device presented in Fig. S5b is lower than that presented in Fig. S5a.

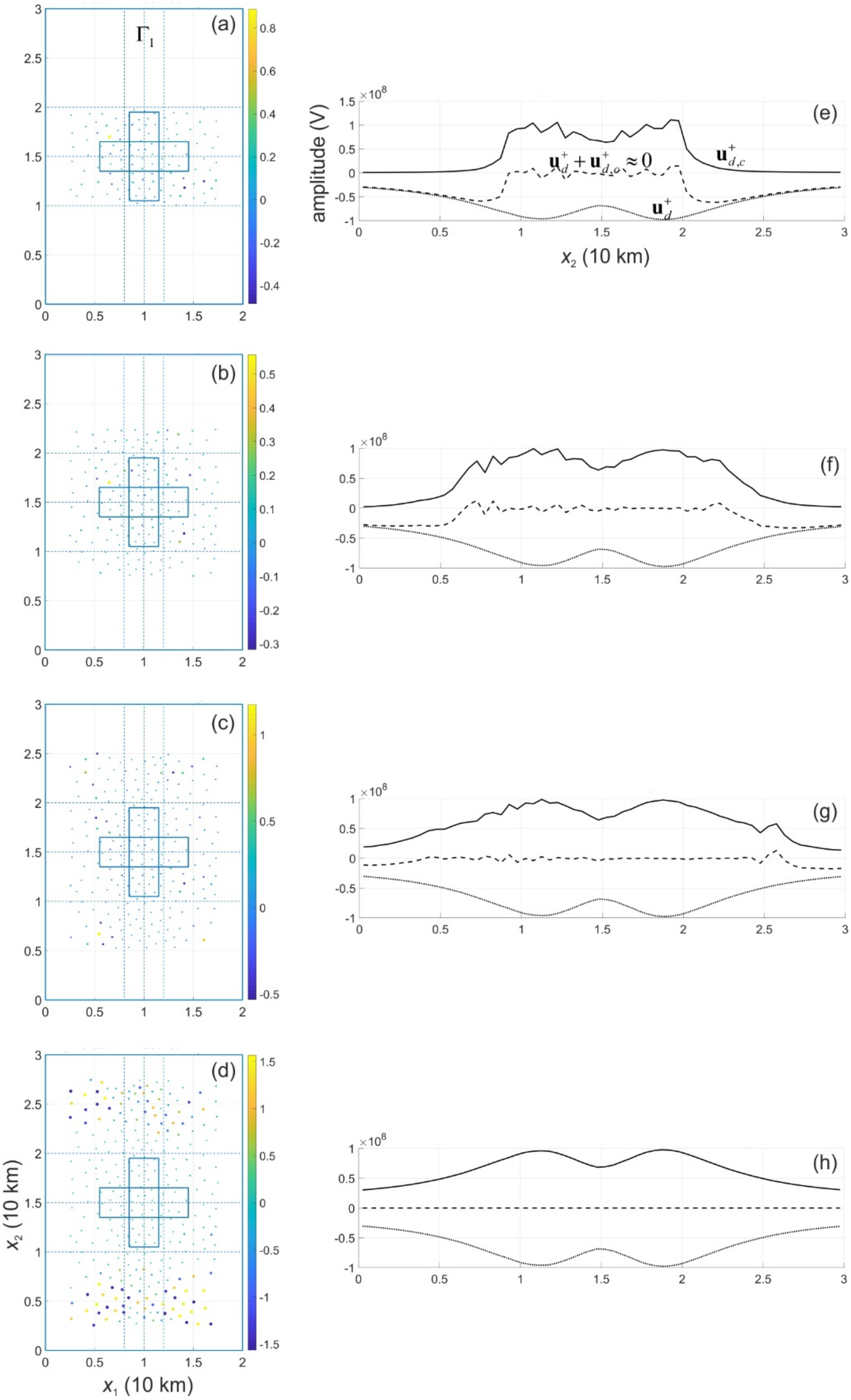

Fig. S2. Efficiency of the cloaking device $\mathbf{f}^{+}_{c,\alpha}$ depending on its size: 15 km x 10 km (a), 15 km x 15 km (b), 15 km x 20 km (c), and 15 km x 25 km (d). The right panels (e-h) present the corresponding cloaking of the electric potential on line $\mathbf{\Gamma}_1$.

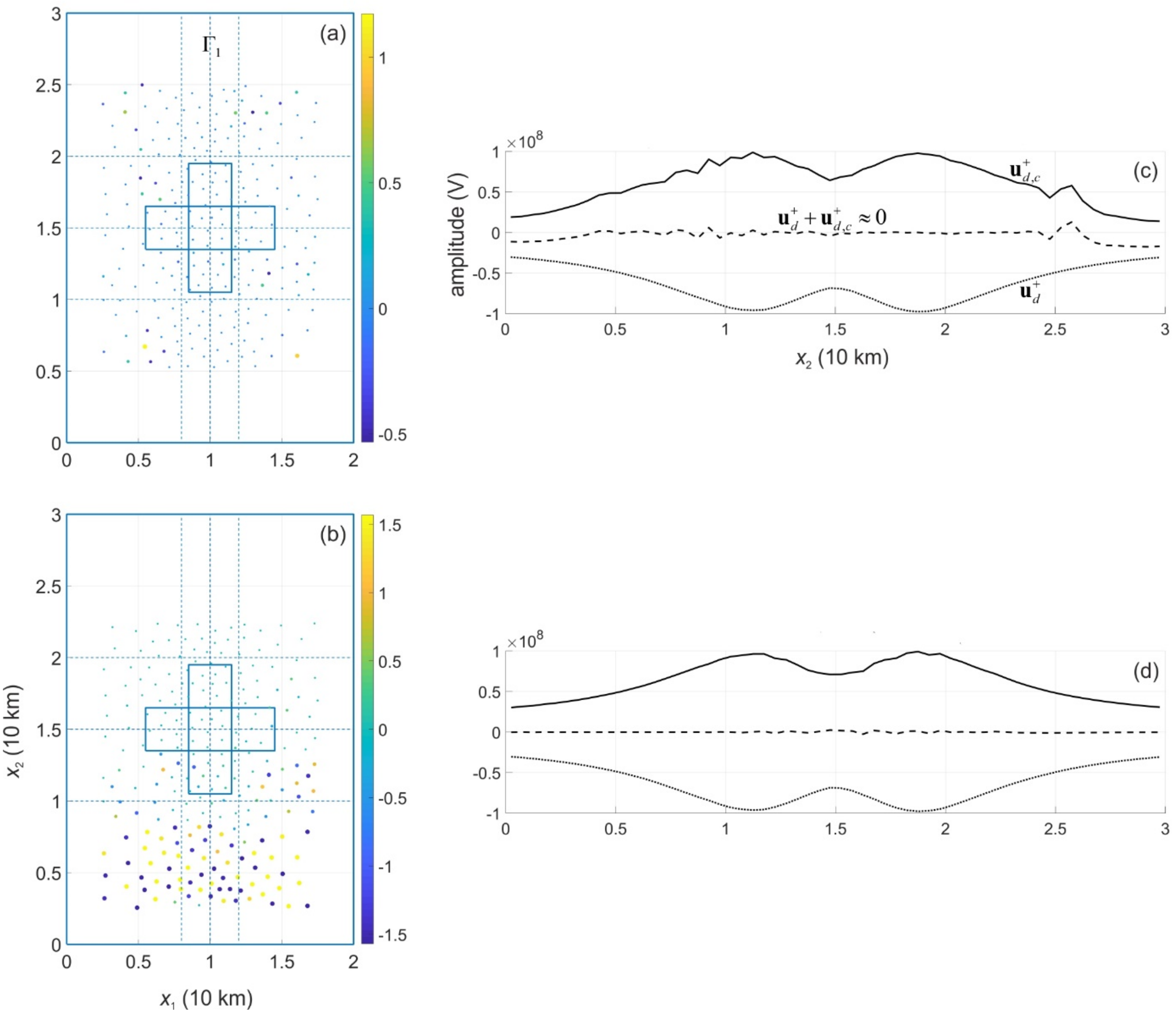

Fig. S3. Efficiency of the cloaking device $\mathbf{f}^{+}_{c,\alpha}$ of size 15 km x 20 km depending on its position: (a) it is located in the centre of the ISD; and (b) it is displaced by 1.5 km toward the south in x_2-direction. Panels (c) and (d) present the corresponding cloaking of the electric potential on line $\mathbf{\Gamma}_1$.

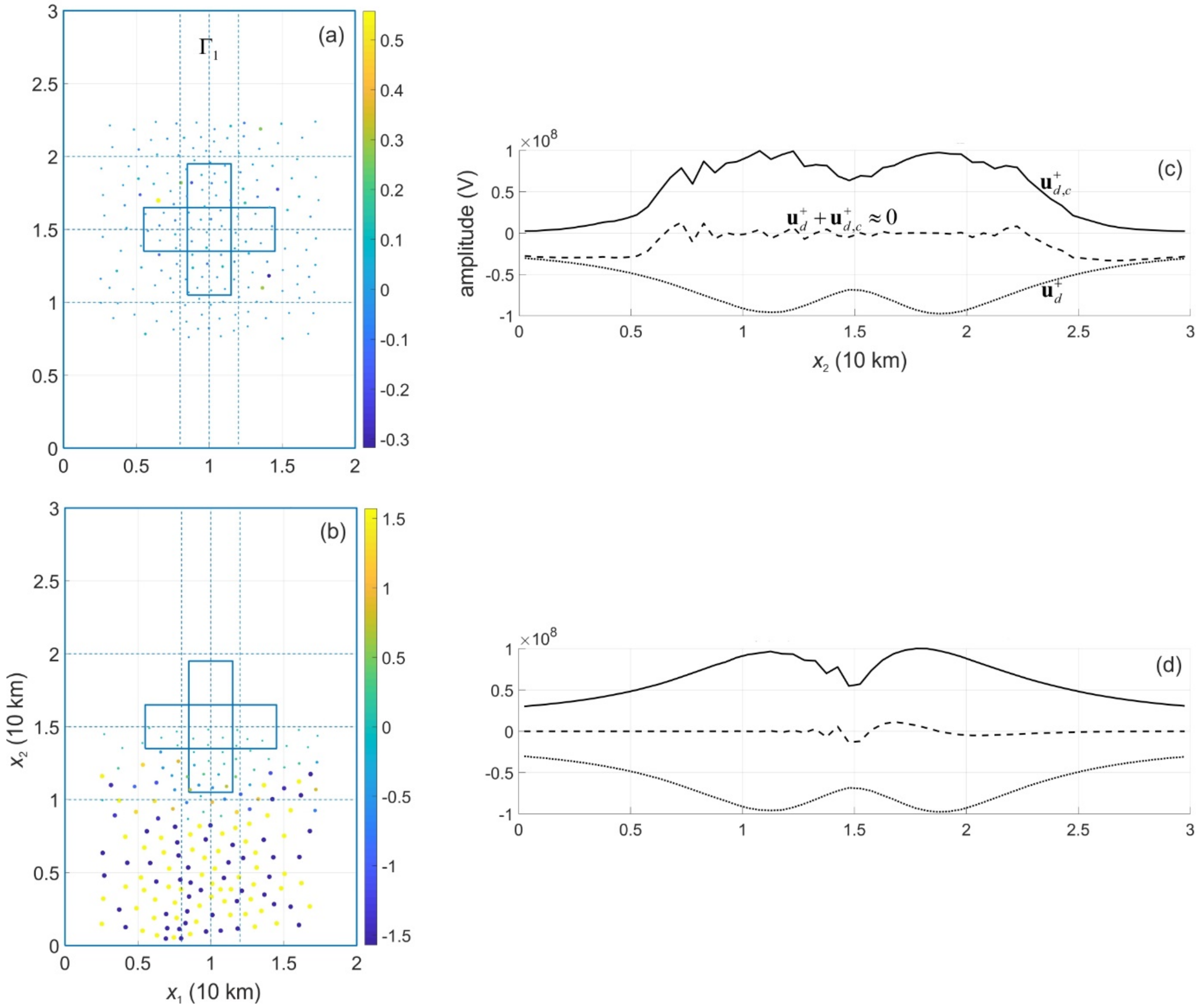

Fig. S4. Efficiency of the cloaking device $\mathbf{f}^{+}_{c,\alpha}$ of size 15 km x 15 km depending on its position: (a) it is located in the centre of the ISD; and (b) it is displaced by 7.5 km toward the south in x_2-direction. Panels (c) and (d) present the corresponding cloaking of the electric potential on line $\mathbf{\Gamma}_1$.

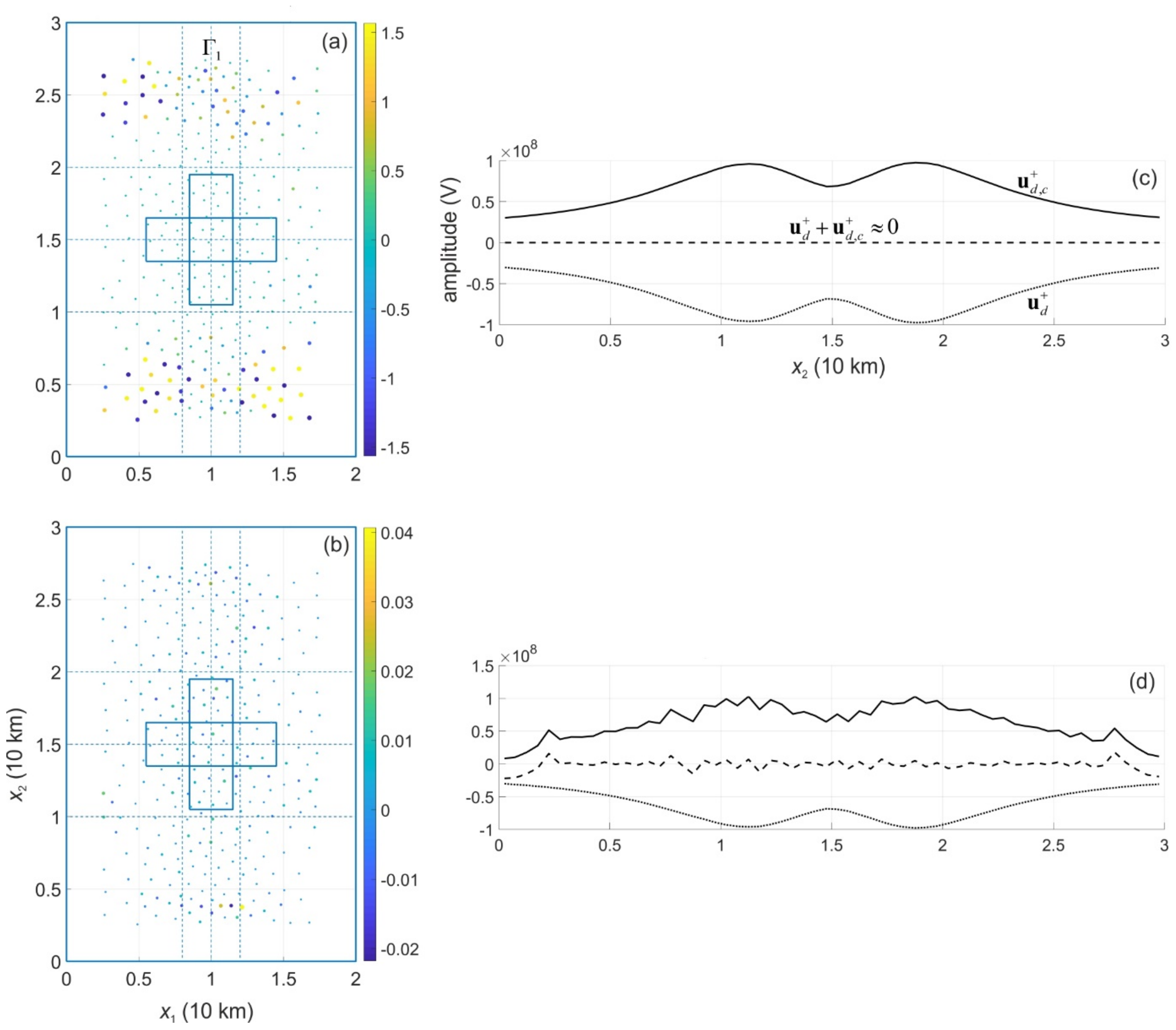

Fig. S5. Cloaking device (a) and its electric potential signal cancellation (c) based on synthetic measurements $\mathbf{u}_d$ along line $\boldsymbol{\Gamma}_1$. Cloaking device (b) and its signal cancellation (d) based on synthetic measurements $\mathbf{u}_d$ taken in the entire OSD.
